# Potential Component Allee Effects and Their Impact on Wetland Management in the Conservation of Endangered Anurans

**DOI:** 10.1371/journal.pone.0010102

**Published:** 2010-04-09

**Authors:** Michele A. Gaston, Akiko Fuji, Floyd W. Weckerly, Michael R. J. Forstner

**Affiliations:** Department of Biology, Texas State University - San Marcos, San Marcos, Texas, United States of America; University of Liverpool, United Kingdom

## Abstract

Effective management of wetland quantity and quality is crucial for effective conservation of declining amphibian populations. In particular, frogs and toads that employ aggregative breeding strategies may suffer negative population impacts in response to changes in availability of aquatic breeding habitat, including overabundance of suitable habitat, if density of conspecifics attending aggregations is positively correlated with reproductive success. Here we document such a positive relationship, potentially the first example of a component Allee effect in an anuran, in the critically endangered Houston toad (*Bufo houstonensis*). We assessed the relationship between mean yearly chorus size and reproductive success of males at the pond level using an information theoretic model selection approach and a two-sample t-test. The chosen model contained the single variable of mean yearly chorus size to predict probability of reproduction, as selected using the Akaike Information Criterion corrected for small sample size and Akaike weight. Mean chorus sizes were significantly higher among ponds exhibiting evidence of reproduction than in those that showed no evidence of reproduction. Our results suggest that chorusing alone is a poor proxy for inference of population stability and highlight a need for reassessment of widely-used amphibian monitoring protocols. Further, amphibian conservation efforts should account for potential Allee effects in order to optimize benefits and avoid underestimating critical population thresholds, particularly in species exhibiting rapid population declines.

## Introduction

Wetland quantity and quality are a primary concern in conservation of amphibians that have an aquatic larval stage [1]. Population dynamics in pond-breeding amphibians are often assumed to follow a metapopulation model [2], wherein local extinction and recolonization at the pond level is common and necessary for long-term stability [3]. Thus, draining of existing wetlands or replacement of ephemeral wetlands by more permanent impoundments (e.g. livestock ponds) may have negative impacts on amphibian populations [1]. Management strategies may call for construction of artificial wetlands to mitigate these negative impacts. While creation of new habitat is intuitively favorable, planning and construction of artificial habitats should be guided by sound empirical evidence in order to maximize benefits [1]. Many amphibians, particularly frogs and toads, employ aggregative breeding strategies which serve to dramatically increase population density at a highly local scale [4]–[6]. Given well-documented amphibian declines [7], [8], the effects of increased breeding habitat availability on aggregation size and overall reproductive output of individual breeding sites merit greater scrutiny than has thus far been empirically documented. Smaller or less dense breeding aggregations may attract fewer females, thereby reducing mating probability for males attending smaller choruses, and may have subsequent negative population impacts. A reduction in mate-finding ability at low population density that has negative effects on individual fitness components and/or per capita population growth rate is an example of the Allee effect [9].

Allee effects are defined as any positive relationship between individual fitness components (component Allee effects) or per capita population growth rate (demographic Allee effects) and population density or overall numbers of conspecifics [9], [10], and are often investigated in organisms demonstrating some degree of sociality [9], [11]. Appreciation for the importance of Allee effects in rare and endangered taxa is increasing, and a number of theoretical models now exist to predict extinction boundaries within species or populations for a variety of demographic characteristics and mating systems [9], [12]. Failure in mate-location is one of the most common examples of the Allee effect and is known to contribute to extinction probabilities in many rare species [9]. Frog and toad species that employ aggregative breeding strategies may be particularly vulnerable to component Allee effects as their populations decline. Previous work in a number of anuran taxa suggest that female attendance at choruses [5], [13] and operational sex ratio [14], [15] depend on chorus size, both of which may impact an individual male's probability of mating [5], [16]. Thus breeding chorus size is one aspect of anuran biology in which component Allee effects may be measured, if the number of males attending choruses and the reproductive output is known. However, Allee effects have yet to be described in amphibians [17] despite ongoing loss of biodiversity in this group [7]. Our research assessed the potential for Allee effects in the mating system of a rare amphibian, the critically endangered Houston toad (*Bufo houstonensis*).

The historic distribution of *B. houstonensis* encompassed 12 counties in east-central Texas [18]. Populations in three of these counties were extirpated by the 1970s, and recent surveys across this range detected toads in only five counties (MRJF unpublished data). Intensive pond level monitoring of reproductive activities (breeding choruses, oviposition, tadpoles, juveniles) has been conducted since 2000 within the critical habitat described by the United States Fish and Wildlife Service in Bastrop County, TX [19]–[22] in concordance with the recovery plan established for this species [22]. We examined whether there is a relationship between the mean number of calling male *B. houstonensis* attending choruses within a breeding season and evidence of reproductive activity at the pond level, and whether there is a minimum threshold of mean yearly chorus size required to observe reproduction. In addition, we assessed whether a statistically significant difference exists between number of males attending choruses that fail to attract females and those that attracted at least one female as indicated by eggs, tadpoles, or juvenile toads.

## Materials and Methods

### *B. houstonensis* Reproductive Ecology

*Bufo houstonensis* is considered an explosively-breeding anuran, with chorusing typically occurring in two to three day pulses intermittently from January through June. Nighttime temperatures above 14°C have been suggested as the most important environmental cue for chorusing [23]. Eggs are deposited in shallow, lentic waters in long strings that may exceed 1000 individual eggs (MRJF personal observation, this study). Tadpoles become free-swimming within two weeks of oviposition and typically remain within several meters of the oviposition site. Time to emergence varies, but is typically 30–45 days after oviposition, and members of an individual cohort undergo metamorphosis within one week (K. Greuter unpublished thesis).

### Study Area and Data Collection

We collected all data at the Griffith League Ranch (GLR) in Bastrop County, Texas. The study site and acoustic survey methods are described in previous publications [19]–[21]. Surveys were conducted on nights when overnight temperatures were forecast to remain above 14°C, and all 17 ponds were surveyed on each survey night. Chorus size was either estimated acoustically (for chorus size ≤5) or obtained by walking the pond edges and counting the number of calling males present. We surveyed 17 ponds from 2000 to 2006 on nights when environmental conditions were appropriate for *B. houstonensis* breeding behavior [23]. Observers trained in the recognition of anuran eggs and tadpoles gathered data on reproductive output. We obtained these data by pond edge surveys during daylight hours, approximately every two weeks throughout the duration of the breeding and emergence season. Given the persistence of eggs, tadpoles, and post-metamorphic toads in and around the natal pond, the probability of detecting reproduction using this method is high. Although there is one other common bufonid species (*Bufo nebulifer*) that is sympatric with *B. houstonensis* at this location, the timing of reproduction is offset in these two species, particularly in observed chorusing behavior [23]. However, metamorphosis and emergence of these two species may overlap. Thus, in cases where species identification was questionable, specific identity was determined using mtDNA and nuclear microsatellite markers (K. Greuter unpublished thesis; D. J. McHenry ongoing dissertation).

Survey effort was limited by the availability of qualified researchers in some years and on some nights, thus in some instances the number of males in large choruses was recorded as >10 rather than counting each male. For this reason we grouped all observed chorus sizes of 10 or larger as a single category (≥10) for analysis. Further, on several occasions (n = 4) evidence of reproduction was observed at ponds where no chorusing (MCS = 0.0) was detected during acoustic surveys at any time during the breeding season. To the best of our knowledge, reproduction in *B. houstonensis* always and by necessity is preceded by chorusing, as is generally true of acoustically-communicating amphibians [5], [6]. Since inclusion of these four individual observations in our analyses would serve only to obfuscate the relationship between chorus size and reproduction, and given our conviction that these observations were obtained in error, we chose to remove them from the dataset prior to data analysis. We attribute these observations to the occasions on which severe weather prevented the safe completion of surveys during conditions that were otherwise suitable for *B. houstonensis* reproduction. Two ponds (pond 4 and pond 17) did not support any evidence of *B. houstonensis* breeding behavior during any survey year and were not included in our analyses.

### Statistical Analyses

Reproductive output was considered as positive or negative (REP+, REP−). We scored a pond REP+ if eggs, tadpoles, or juvenile toads were observed at the pond at any point during the reproductive season of a given year. We calculated mean yearly chorus size (MCS) as the total number of males heard calling at a pond through the entire breeding season divided by the number of surveys during the season. To determine if a relationship exists between MCS and REP, we employed an information theoretic model selection approach. The Akaike Information Criterion corrected for small sample size (AIC_c_) [24] and Akaike weights were used to choose the model that best fit the observed reproductive events. MCS was coded as a continuous, fixed predictor for model fitting. In order to assess the potential influence on observed reproduction of pond quality and climatological variation among years, we included terms for pond and year in the model fitting process. Both variables were treated as fixed categorical predictors. We used the chosen model to calculate the probability of reproduction across the empirical range of MCS.

Owing the nature of *B. houstonensis* breeding phenology (explosive breeding with sporadic chorusing events through late winter and spring), the MCS dataset contains many survey nights on which no toads were heard calling. Therefore, while MCS is a useful variable for assessing component Allee effects at the level of the pond, this value may significantly underestimate the actual number of calling males necessary for successful reproduction. To further understand the effect of chorus size on reproductive success, we removed the 45 MCS values that represented years during which no chorusing was detected at a pond, and calculated mean chorus size (CHOR) from only the nights on which at least one male was heard calling for each pond and year. These values were then divided into two groups based on whether reproduction was observed (CHOR+) or not (CHOR−), and the distributions compared using a two-sample t-test. CHOR values were ln transformed prior to conducting the test to meet the assumption of homoscedasticity.

## Results

### Qualitative Indicators

Our analyses included 1,935 survey nights after exclusion of surveys of ponds where reproduction occurred with no chorusing detected during that year. Chorusing was detected on 139 survey nights, or in 7.0% of all surveys. This number reflects the breeding phenology of *B. houstonensis*, an explosive breeder that typically breeds on only a few nights during the spring in response to environmental conditions that favor reproduction [23]. Chorusing was detected at 55.1% of ponds surveyed across all years, eggs and/or tadpoles were detected at 19.9% of ponds surveyed across all years, though emergence was detected at only 7.8% of ponds surveyed across all years (Table 1). A total of 101 MCS values were included in our analyses, of which 45 MCS values were 0 (i.e. no chorusing was detected at that pond during that survey year). MCS, CHOR, number of nights surveyed, and number of nights on which at least one male *B. houstonensis* was detected calling are summarized for each pond and year in Table S1.

**Table 1:** Frequency of observed Houston toad (*Bufo houstonensis*) chorusing, eggs or tadpoles, and emergence for each year and across all years for 17 ponds in Bastrop County, TX.

	2000	2001	2002	2003	2004	2005	2006	Overall
Chorusing	0.333	0.643	0.933	0.462	0.533	0.667	0.286	0.551
Eggs/Tads	0.267	0.500	0.200	0.154	0.000	0.200	0.071	0.199
Emergence	0.067	0.143	0.133	0.000	0.000	0.133	0.071	0.078

**Figure 1:**
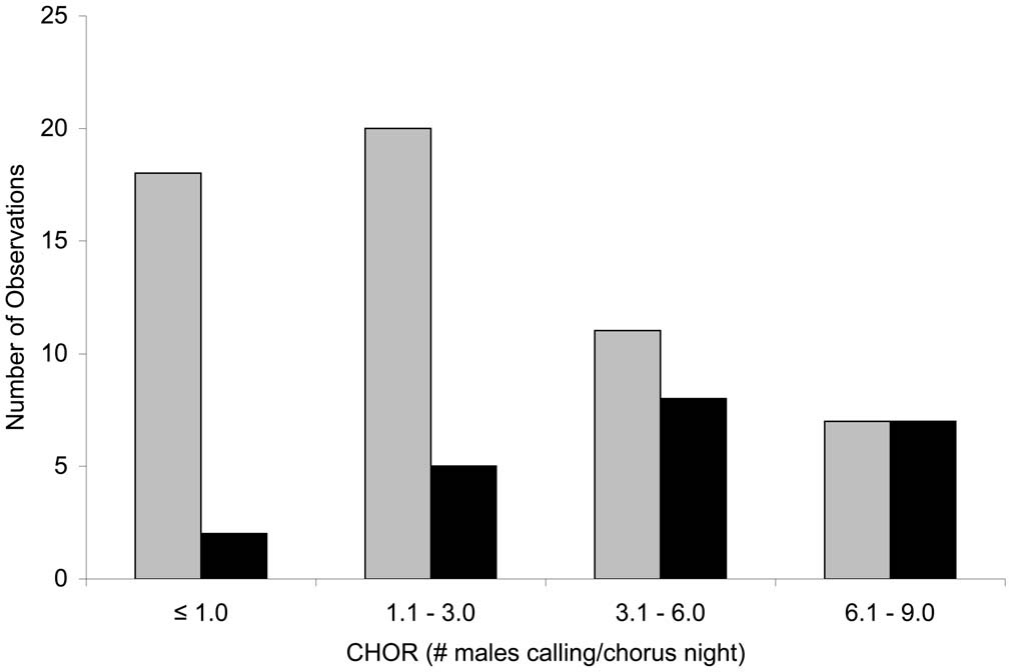
Frequency of observation and reproductive success of four chorus size classes. Mean number of Houston toad (*Bufo houstonensis*) males attending choruses calculated from nights on which at least one male was heard calling (CHOR, grey bars) and number of ponds exhibiting evidence of reproduction (black bars) for four chorus size classes. Proportion of ponds with evidence of reproduction goes up as CHOR increases, with all ponds in the largest size class having evidence for reproduction.

In order to further assess the trend in the relationship between chorus size and reproduction, we generated a frequency histogram of mean chorus sizes and observed reproduction (Fig. 1). We used means calculated from only nights on which at least one male was heard calling (CHOR). Size ranks were ≤1.0, 1.1–3.0, 3.1–6.0, and ≥6.0. The boundaries of the classes were chosen based roughly on natural breaks in the CHOR dataset. The proportion of ponds with reproduction observed steadily increased with increasing chorus size, and all ponds showed evidence of reproduction in the largest size class.

### Model Fitting and Regression Analysis

**Figure 2:**
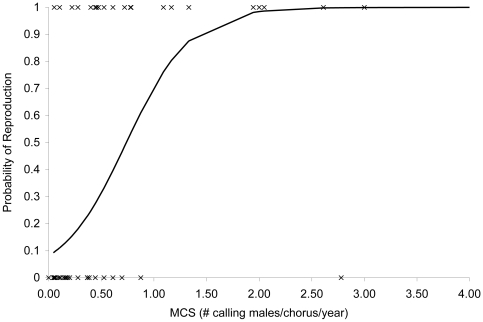
Probability of Houston toad (*Bufo houstonensis*) reproduction as a function of mean chorus size. X = observed reproduction. Line represents probability of reproduction as predicted using the logistic regression model [P(REP) = e ^(−2.43+3.29*MCS)^/1+e ^(−2.43+3.29*MCS)^]. Probability of reproduction approaches 100% at mean yearly chorus size of 2.0 males.

The model that best fit the observed data contained a predictor for MCS only. Pond and year were not significant predictors of observed reproductive activity in any of the model evaluation iterations. We were unable to evaluate interactions between predictors because of insufficient degrees of freedom (see Table 2 for the models considered, AIC_C_ values, and Akaike weights). Since the chosen model contained a single, continuous predictor for a two-category response variable (REP+, REP−), this model was a simple logistic regression equation. We found that there is a significant, positive relationship between MCS and probability of reproduction (REP = −2.43+3.29*MCS; P = .00014; z = 3.801). We used the logistic regression equation to calculate expected probabilities of reproduction and the results were plotted against MCS for values ranging from 0 to 4 (Fig. 2). At MCS of approximately 2 males calling per pond per year, the graph approaches 100% probability of observing evidence of reproductive activity. Thus ponds having MCS of less than 2 males are less likely to support reproduction, and the probability of successful breeding decreases as MCS decreases.

**Table 2:** Models evaluated, −2 log likelihood [−2(LL)], k, Akaike information criterion corrected for small sample size (AICc), and Akaike weights used in model fitting procedure testing chorus size effects on observed reproduction (rep) for the Houston toad (*Bufo houstonensis*).

Model	−2(LL)	k	AICc	Akaike wt
p(rep) = b_0_	105.88	1	107.97	1.34^−07^
p(rep) = b_0_+b_1_(MCS)	72.40	2	76.45	0.938
p(rep) = b_0_+b_1_(year)	98.32	8	113.89	6.96^−09^
p(rep) = b_0_+b_1_(pond)	73.07	16	109.55	6.09^−08^
p(rep) = b_0_+b_1_(MCS)+b_2_(pond)	58.09	17	97.38	2.67^−05^
p(rep) = b_0_+b_1_(MCS)+b_2_(year)	63.95	9	81.87	6.24^−02^
p(rep) = b_0_+b_1_(pond)+b_2_(year)	61.65	23	117.99	8.95^−10^
p(rep) = b_0_+b_1_(MCS)+b_2_(pond)+b_3_(year)	49.05	24	108.78	8.95^−08^

The model that included mean yearly chorus size (MCS) had a 0.94 probability of being the best model as measured by Akaike weight.

### Comparison of Mean Chorus Size

The means of the CHOR+ and CHOR− distributions differed significantly as assessed by the two-sample t-test (mean_CHOR+_ = 5.245, n = 22; mean_CHOR−_ = 1.869, n = 34; P = 1.149^−06^). Note that the mean for reproduction-positive ponds represents a conservative estimate, as chorus sizes of 10 or more males were grouped to account for variation in the sampling approach (counting males at the pond edge vs. estimating chorus size acoustically). Further, of 21 individual choruses of 10 or more individuals, four (19.1%) had no evidence of reproduction, suggesting that mean CHOR for reproductively successful ponds is likely numerically higher than indicated by our results.

## Discussion

Our qualitative results and statistical analyses reveal a need for careful assessment of potential Allee effects in declining amphibian populations, and for broad reexamination of the utility of acoustic chorus survey data as a proxy for reproduction. The qualitative results indicate that, for this endangered toad species, detection of chorusing is not a suitable proxy for reproduction. Acoustic chorus surveys are one of the most common methods for monitoring of amphibians [e.g. 25], [26], [27] and their use in population trend assessment implicitly regards chorusing to act as a proxy of reproduction. Our results contradict that paradigmatic assumption, in that the proportion of ponds with chorusing detected far exceeds the proportion of ponds with evidence of reproduction, particularly emergence (Table 1). While we did not systematically sample for female toads during this study, when female toads were encountered at choruses, they were more often present at larger choruses. This suggests that the observed relationship between reproduction and chorus size could result from a failure of females to move toward breeding sites where few males are calling. Alternatively, predator satiation could explain our observations, if only a few females oviposit at small-chorus sites and all eggs or tadpoles are subsequently consumed. While predator/prey interactions were likely important in determining emergence in ponds where eggs were detected but later life stages were not, we believe it is extremely likely that lack of females attending small choruses is the mechanism underlying our failure to detect any evidence of reproduction at ponds having low MCS values. Our conviction is based on a lack of evidence of direct predation on observed egg strings during the course of this research coupled with the unanalyzed trend for females to be present at ponds where larger numbers of males are calling.

Statistically, the results of both analyses indicate that larger choruses have a higher probability of successful reproduction. We interpret these results in light of an individual male's probability of mating: males attending smaller choruses experience decreased probability of successfully reproducing, averaged within a chorus, as measured by presence or absence of eggs, tadpoles, or metamorphic toads. Stated differently, males attending smaller choruses experience reduced average fitness and may be experiencing a component Allee effect. While a direct measure of individual male fitness (e.g number of clutches fertilized) is needed to conclusively demonstrate a component Allee effect, our results strongly suggest that a component Allee effect may exist, and that individual males attending smaller choruses likely have a very low probability of producing offspring. Conclusive evidence for component Allee effects requires further research.

While our study did not address underlying causes of the observed relationship between chorus size and reproductive success, several hypotheses could explain our observations. First, sex ratios in *B. houstonensis* are male-biased [21]. Thus overall toad density in a given habitat patch could be so low that no females are within an acoustically-detectable distance of a breeding chorus. While direct measurement of toad density in upland or riparian habitats is difficult, an ongoing mark-recapture study using drift fence arrays and breeding chorus sampling should help in estimating that density in the near future. Second, habitat degradation in the form of severely overgrown woody understory may impede sound transmission [28] and effectively reduce the distance over which choruses are detectable to *B. houstonensis*. Further research is needed to determine the distance over which *B. houstonensis* calls can be heard in different habitat structures (e.g open understory vs. overgrown understory), and the effect of chorus size on those distances. Habitat interference with signal transmission could affect the ability of either gender to perceive choruses, and could thus interfere with the underlying mechanisms of chorus formation. Third, sexual selection favoring larger choruses, operating on preferences of either sex (or both), could produce the observed threshold in reproductively-successful chorus size. Few studies have investigated sexual selection for chorus size in anurans [14], [16], and conclusions are inconsistent. Should either male or female toads exhibit a positive phonotactic response to larger choruses when offered a choice between small and large choruses, our observations could reflect the end result of sexual selection. This would suggest that chorus formation is influenced by factors in addition to overall toad density in a given habitat patch (first hypothesis), though the interaction of low density and sexual selection favoring large choruses could lead to increasing reproductive failure at the level of individual breeding ponds. Finally, reproductive asynchrony in arrival at the breeding site between male and female toads could produce the observed result, if male toads abandon the breeding site before females arrive [23], [29]. The above-mentioned research should help determine the timing of female arrival at breeding sites relative to the onset of chorusing.

These hypothetical causes are not mutually exclusive and interactions among them could exist in *B. houstonensis* and many other anurans, thus both the explicit and potentially interacting proximal and ultimate causes of this relationship are not clear today. Research is either planned or under way to elucidate these causes and the interactions among them. The summative causes will ultimately reflect the evolutionary history of this species, and consequent adaptive thresholds to anthropogenic changes to the environment.

Evolutionary and ecological history of a species may be particularly important when assessing the presence of Allee effects and in development of effective management strategies. Theory predicts greater susceptibility to Allee effects in organisms that historically existed in high densities but that have become rare when compared to organisms that have a history of low population density [9]. In the latter group, these theoretical models predict adaptations that compensate for any fitness consequences that result from impaired mate-finding and that the population will persist at a stable (though low) density. Early field research on breeding dynamics of *B. houstonensis* [23], [30] strongly suggest that it belongs in the former category, and that small breeding choruses observed over the past ten years represent a dramatic deviation from long-term norms for this species [23], [30], [31]. Since observed chorus sizes are now on average an order of magnitude smaller than a decade to three decades ago [23], [30], *B. houstonensi*s serves as a particularly relevant anuran species for evaluation of Allee effects generally.

One of the more prominent ecological changes within the range of *B. houstonensis* is the addition of numerous permanent livestock tanks over the past 100 years. Prior to this period, available breeding habitat was likely limited to ephemeral ponds that persisted for short periods of time, with no or extremely few permanent lentic habitats. Thus both quantity and quality (particularly hydroperiod) of current breeding habitat is dramatically different from what existed during the entire evolutionary history of this species. Increased density and permanence of ponds may represent an evolutionary hurdle to *B. houstonensis*, particularly in the face of population declines, in that breeding behaviors shaped by ecological and sexual selection over many thousands of years may be maladaptive in the current environment. Specifically, increasing pond density and decreasing toad density implies that fewer individual males will attend choruses at more individual ponds, and thereby holds the potential to negatively impact reproduction range-wide. We hypothesize that habitat degradation, sexual selection in either sex for larger choruses, or both, could exacerbate this negative effect of increasing pond density. Given population trends through the years since *B. houstonensis* was described, and the implications of our results, we speculate that component Allee effects may exist in this species, and therefore recommend an immediate moratorium on pond construction within the existing forested range of *B. houstonensis*.

The relationship we described may represent the first documented example of an Allee effect in an amphibian. We consider this as a component Allee effect (i.e. positive density-dependent individual fitness) rather than a demographic Allee effect because our study did not directly address the role of chorus size on overall population growth rate. However, chorus sizes measured in the present study and in ongoing rangewide acoustic surveys are often two orders of magnitude smaller than the largest choruses reported from several decades ago [23], [30]. Further, rangewide acoustic surveys document the apparent loss of *B. houstonensis* from three additional counties in the past twenty years [32]–[35]. What we consider as a possible component Allee effect may in fact contribute to rangewide population declines and may thus contribute to a demographic Allee effect (i.e. positive density-dependent per-capita population growth rate). Theory predicts that mating strategies that reduce fitness should have an effect on population growth rate [29], though theoretical studies demonstrating this relationship far outnumber those that demonstrate this relationship empirically [9], [29].

*Bufo houstonensis* exhibits an explosive breeding phenology common to many anuran species across the globe [5], [6]. The Allee effect that could be operating in this species may well exist in other anurans with similar reproductive behaviors, which have experienced similar sexual and ecological selection pressures leading to convergence in reproductive behavior, and should be most detectable in declining populations. Given the current threats to biodiversity within Amphibia [36], Allee effects merit more extensive investigation within this group. Failure to include Allee effects in population models when they are present can lead to underestimates of extinction thresholds [9], and ultimately failure of conservation measures to fully address threats to declining populations. The methods we employed used data commonly collected in anuran population surveys, and thus provide a means for rapid assessment of potential Allee effects in actively monitored anuran species.

It is unclear at present how common component Allee effects may be in acoustically communicating anurans. Should additional research show this to be a widespread phenomenon, call survey data for endangered or threatened species should be carefully reexamined for consistently small chorus sizes and for trends toward smaller chorus sizes. Species and/or populations exhibiting these characteristics may be at higher risk of extinction than has previously been assumed, and should be targeted for efforts aimed at increasing density at individual breeding sites. Conversely, areas within a given species' range that consistently support large choruses represent optimal targets for long-term conservation. In addition, management strategies that call for adding aquatic breeding habitat for anurans should be reevaluated with potential consequences resulting from undetected Allee effects in mind.

## Supporting Information

Table S1 Data used in t-test, model fitting, and logistic regression analyses.
(0.14 MB DOC)
