# Supplementary material for: Potential Component Allee Effects and Their Impact on Wetland Management in the Conservation of Endangered Anurans
Source: PLoS One. 2010 Apr 9;5(4):e10102. doi: 10.1371/journal.pone.0010102 (PMC2852421; doi:10.1371/journal.pone.0010102)
Supplement: Table S1 — Data used in t-test, model fitting, and logistic regression analyses. (0.14 MB DOC) [file pone.0010102.s001.doc]

| Pond |  | 2000 |  |  | 2001 |  |  | 2002 |  |  | 2003 |  |
| --- | --- | --- | --- | --- | --- | --- | --- | --- | --- | --- | --- | --- |
|  | MCS | CHOR | n (n_CHOR_) | MCS | CHOR | n (n_CHOR_) | MCS | CHOR | n (n_CHOR_) | MCS | CHOR | n (n_CHOR_) |
| 1 | 0.00 | 0.00 | 11(0) | 0.00 | 0.00 | 18(0) | 0.17 | 3.00 | 18(1) | 0.00 | 0.00 | 19(2)** |
| 2 | 2.00 | 7.33 | 11(3)* | 1.94 | 8.75 | 18(4)* | 2.61 | 6.71 | 18(7)* | 2.00 | 7.60 | 19(5)* |
| 3 | 0.00 | 0.00 | 11(0) | 0.06 | 1.00 | 18(1) | 0.00 | 0.00 | 18(0) | 0.00 | 0.00 | 19(0) |
| 5 | 0.00 | 0.00 | 11(0) | 1.33 | 4.80 | 18(5)* | 1.17 | 2.63 | 18(8)* | 0.11 | 1.00 | 19(2) |
| 6 | 0.46 | 2.50 | 11(2)* | 0.22 | 4.00 | 18(1) | 0.17 | 1.50 | 18(2) | 0.05 | 1.00 | 19(1)* |
| 7 | 0.00 | 0.00 | 11(0)** | 0.61 | 5.50 | 18(2)* | 0.44 | 4.00 | 18(2)* | 0.11 | 1.00 | 19(2) |
| 8 | 0.00 | 0.00 | 11(0)** | 0.11 | 1.00 | 18(2) | 0.11 | 1.00 | 18(2) | 0.00 | 0.00 | 19(0) |
| 9 | 0.00 | 0.00 | 11(0) | 0.78 | 3.50 | 18(5)* | 0.44 | 4.00 | 18(2) | 0.05 | 1.00 | 19(1) |
| 10 | 0.00 | 0.00 | 11(0) | 0.72 | 6.50 | 18(2)* | 0.28 | 1.67 | 18(3) | 0.00 | 0.00 | 19(0) |
| 11 | 0.18 | 2.00 | 11(1) | 0.28 | 5.00 | 18(1)* | 2.78 | 2.50 | 18(2) | 0.00 | 0.00 | 19(0)** |
| 12 | 0.09 | 1.00 | 11(1) | 0.00 | 0.00 | 18(0) | 0.61 | 2.75 | 18(4) | 0.37 | 2.33 | 19(3) |
| 13 | 0.00 | 0.00 | 11(0) | 0.00 | 0.00 | 18(0) | 0.17 | 1.00 | 18(3) | 0.00 | 0.00 | 19(0) |
| 14 | 0.00 | 0.00 | 11(0) | 0.00 | 0.00 | 18(0) | 0.06 | 1.00 | 18(1) | 0.00 | 0.00 | 19(0) |
| 15 | 0.00 | 0.00 | 11(0) | 0.00 | 0.00 | 18(0) | 0.20 | 1.33 | 18(3) | 0.00 | 0.00 | 19(0) |
| 16 | 1.09 | 4.00 | 11(3)* | 0.00 | 0.00 | 18(0) | 0.28 | 2.50 | 18(2) | 0.00 | 0.00 | 19(0) |
| Total |  |  | 165(10) |  |  | 270(23) |  |  | 270(42) |  |  | 285(14) |

Table S1: Data used in t-test, model fitting, and logistic regression analyses.

Mean yearly chorus size (MCS), mean chorus size of nights on which at least one *Bufo houstonensis* was heard calling (CHOR), number of nights surveyed (n), and number of nights with at least one male *B. houstonensis* detected calling (n_CHOR_, in parentheses) for all ponds surveyed from 2000 through 2006.

* = ponds having evidence of reproduction (eggs, tadpoles, metamorphs).

** = ponds with reproduction detected but MCS = 0 (excluded from analyses).

Table S1 (continued)

| Pond |  | 2004 |  |  | 2005 |  |  |  | 2006 |
| --- | --- | --- | --- | --- | --- | --- | --- | --- | --- |
|  | MCS | CHOR | n (n_CHOR_) | MCS | CHOR | n (n_CHOR_) | MCS | CHOR | n (n_CHOR_) |
| 1 | 0.11 | 1.00 | 19(2)* | 0.00 | 0.00 | 21(0) | 0.05 | 1.00 | 23(1) |
| 2 | 0.53 | 2.50 | 19(4)* | 2.05 | 8.60 | 21(5)* | 0.70 | 5.33 | 23(3) |
| 3 | 0.00 | 0.00 | 19(0) | 0.00 | 0.00 | 21(0) | 0.00 | 0.00 | 23(0) |
| 5 | 0.16 | 1.50 | 19(2) | 0.37 | 2.33 | 21(3) | 0.00 | 0.00 | 23(0) |
| 6 | 0.47 | 9.00 | 19(1)* | 0.07 | 1.00 | 21(1) | 0.00 | 0.00 | 23(0) |
| 7 | 0.05 | 1.00 | 19(1) | 0.39 | 5.00 | 21(1) | 0.00 | 0.00 | 23(0) |
| 8 | 0.00 | 0.00 | 19(0) | 0.14 | 1.50 | 21(2) | 0.00 | 0.00 | 23(0) |
| 9 | 0.16 | 3.00 | 19(1) | 0.53 | 2.50 | 21(4) | 0.05 | 1.00 | 23(1) |
| 10 | 0.00 | 0.00 | 19(0) | 0.00 | 0.00 | 21(0) | 0.00 | 0.00 | 23(0) |
| 11 | 0.00 | 0.00 | 19(0) | 0.61 | 2.75 | 21(4)* | 0.00 | 0.00 | 23(0) |
| 12 | 0.05 | 1.00 | 19(1) | 0.88 | 2.80 | 21(5) | 0.78 | 6.00 | 23(0) |
| 13 | 0.00 | 0.00 | 19(0) | 0.00 | 0.00 | 21(0) | 0.00 | 0.00 | 23(3)* |
| 14 | 0.00 | 0.00 | 19(0) | 0.00 | 0.00 | 21(0) | 0.00 | 0.00 | 23(0) |
| 15 | 0.00 | 0.00 | 19(0) | 0.40 | 2.67 | 21(3)* | 0.00 | 0.00 | 23(0) |
| 16 | 0.05 | 1.00 | 19(1) | 0.09 | 1.00 | 21(1) | 0.00 | 0.00 | 23(0) |
| Total |  |  | 285(13) |  |  | 315(29) |  |  | 345(8) |

Mean yearly chorus size (MCS), mean chorus size of nights on which at least one *Bufo houstonensis* was heard calling (CHOR), number of nights surveyed (n), and number of nights with at least one male *B. houstonensis* detected calling (n_CHOR_, in parentheses) for all ponds surveyed from 2000 through 2006.

* = ponds having evidence of reproduction (eggs, tadpoles, metamorphs).

** = ponds with reproduction detected but MCS = 0 (excluded from analyses).
